# Dispatch of a helicopter emergency medicine service to patients with a sudden, unexplained loss of consciousness of medical origin

**DOI:** 10.1186/s12873-020-00388-x

**Published:** 2020-11-25

**Authors:** J. Mohindru, J. E. Griggs, R. de Coverly, R. M. Lyon, E. ter Avest, on behalf of Air Ambulance Trust Kent Surrey Sussex

**Affiliations:** 1 Air Ambulance Kent, Surrey and Sussex, Redhill Airfield Redhill Aerodrome, Redhill, Surrey, RH1 5YP UK; 2 University of Surrey, Duke of Kent Building, Guildford, School of Health Sciences, Guildford, GU2 7XH UK; 3 Department of Emergency Medicine, University Medical Center Groningen, University of Groningen, Groningen, The Netherlands

**Keywords:** Loss of consciousness, Dispatch, Helicopter emergency medical service

## Abstract

**Background:**

Sudden loss of consciousness (LOC) in the prehospital setting in the absence of cardiac arrest and seizure activity may be a challenge from a dispatcher’s perspective: The aetiology is varied, with many causes being transient and mostly self-limiting, whereas other causes are potentially life threatening. In this study we aim to evaluate the dispatch of HEMS to patients with LOC of medical origin, by exploring to which patients with a LOC HEMS is dispatched, which interventions HEMS teams perform in these patients, and whether HEMS interventions can be predicted by patient characteristics.

**Methods:**

We performed retrospective cohort study of all patients with a reported unexplained LOC (e.g. not attributable to a circulatory arrest or seizures) attended by the Air Ambulance Kent, Surrey & Sussex (AAKSS), over a 4-year period (July 2013–December 2017). Primary outcome was defined as the number of HEMS-specific interventions performed in patients with unexplained LOC. Secondary outcome was the relation of clinical- and dispatch criteria with HEMS interventions being performed.

**Results:**

During the study period, 127 patients with unexplained LOC were attended by HEMS. HEMS was dispatched directly to 25.2% of the patients, but mostly (74.8%) on request of the ground ambulance crews. HEMS interventions were performed in 65% of the patients (Prehospital Emergency Anaesthesia 56%, hyperosmolar therapy 21%, antibiotic/antiviral therapy 8%, vasopressor therapy 6%) and HEMS conveyed most patients (77%) to hospital. Acute neurological pathology was a prevalent underlying cause of unexplained LOC: 38% had gross pathology on their CT-scan upon arrival in hospital. Both GCS (r = − 0.60, *p* < .001) and SBP (*r* = 0.31, *p* < .001) were related to HEMS interventions being performed on scene. A GCS < 13 predicted the need for HEMS interventions in our population with a sensitivity of 94.9% and a specificity 75% (AUC 0.85).

**Conclusion:**

HEMS dispatchers and ambulance personnel are able to identify a cohort of patients with unexplained LOC of medical origin who suffer from potentially life threatening (mainly neurological) pathology, in whom HEMS specific intervention are frequently required. Presenting GCS can be used to inform the triage process of patients with LOC at an early stage.

**Supplementary Information:**

The online version contains supplementary material available at 10.1186/s12873-020-00388-x.

## Background

Up to 50% of the general population will experience an episode of transient loss of consciousness (LOC) of medical origin at some point during their life [1, 2]. The aetiology of LOC is varied, with some causes being transient and mostly self-limiting (such as reflex syncope or orthostatic hypotension), whereas other causes, such as intracranial bleeds or cardiac arrest, are potentially life threatening, and require extensive medical treatment [3, 4].

Emergency medical services (EMS) frequently attend patients with a sudden LOC [5, 6]. Cardiac arrest and seizures are amongst the most prevalent causes of LOC attended by EMS, and emergency medical dispatchers in most EMS systems have an established pathway during the 112/999 call to discern if one of these conditions is present. Determining other causes of LOC is more of a challenge, and often requires examination of the patient by ground ambulance crews [7, 8].

Critical care teams such as Helicopter Emergency Medical Services (HEMS) can be tasked to patients with an unexplained LOC [8], either directly or on request of the crews. HEMS is potentially able to deliver specific advanced interventions to address the cause of the LOC and to support vital medical interventions, ground ambulance clinicians are unable to provide. Further, HEMS involvement has been shown to shorten scene times for critically ill patients [9], and expedite transport times to hospital. A recent study demonstrated that expedited transport by HEMS may save lives in rural areas for non-trauma patients [10]. Further, clinical decision making skills of HES teams may contribute to better referral, reducing the risk of subsequent interfacility transport (and resultant delays in final treatment) [11]. However, HEMS is a scarce resource, and cannot respond to all patients with LOC. Appropriate triage of patients with LOC by dispatchers and ambulance crews is therefore of utmost importance.

In this study we aim to evaluate the dispatch of HEMS to patients with LOC of medical origin, by exploring to which patients with a LOC HEMS is dispatched, which interventions HEMS teams perform in these patients, and whether HEMS interventions can be predicted by patient characteristics.

## Methods

### Study setting and design

A retrospective study was performed of all patients with a LOC who were attended by the Air Ambulance Kent, Surrey & Sussex (AAKSS) between 2 July 2013 and 19 December 2017. AAKSS is a HEMS covering three counties in the southeast of England with a resident population of 4.5 million and transient population of up to 8 million. Two doctor-paramedic teams respond in either a helicopter or response car from one base. The service attends approximately 2000 patients per year. Most patients attended to by the HEMS service are first seen by a ground ambulance crew and/or a critical care paramedic. Ground ambulances in the HEMS catchment area are staffed by paramedics and/or emergency technicians.

The AAKSS HEMS team (consisting of two pilots, a paramedic and a doctor) is dispatched by a dedicated AAKSS dispatcher who is present in the South East Coast Ambulance Service Trust (SECAmb) Emergency Operation Center (EOC) and continuously screens in-coming emergency calls. HEMS dispatchers screen all the incoming calls on the CAD system of the ambulance service (SeCamb). Secamb uses NHS pathways, which is a well-structured, evidence based system that uses symptom descriptions to guide call handlers along robust clinical decision trees. As the Emergency Operations Centre (EOC) handles more than one million calls every year, HEMS dispatchers are particularly focused on calls with a predicted cat 1 or 2 dispatch of the ambulance service (absent-or abnormal breathing, or unconscious) based on the nature of call (NoC) screening prior to full triage.

AAKSS dispatchers have a background of ambulance dispatch, with extensive experience of working in the ambulance control room. They are aided by a bespoke tasking algorithm, devised by the AAKSS management team (Supplemental file 1) [12]. Whilst listening to the incoming emergency call, dispatchers aim to rapidly identify either one (from Grade 1 criteria list) or two (from Grade 2 criteria list) dispatch criteria. If these are positively identified, HEMS is dispatched immediately. Furthermore, HEMS can be dispatched on request of the critical care desk (CCD) (Grade 3) or on request of the crews on scene with the patient (Grade 4).

### Study population

Patients were eligible for participation in the study if they had sustained a sudden drop in postural tone (collapse) with a LOC that was *not* attributable to a circulatory arrest or seizures according to the attending HEMS team at the time of presentation. LOC classification was documented as a mandatory entry in the bespoke electronic patient clinical record system which AAKSS uses (HEMSbase 2.0, Medic One Systems Ltd., UK). Classifications were reviewed by one of the investigators (JM) and compared against clinical information (including any available post mortem reports and follow-up notes) available in HEMSbase. In case of disagreement with the classification as given by the treating physician, the case was discussed with a second reviewer in order to establish final classification.

### Data collection

The following data were retrieved from the HEMSbase electronic patient record: patient identification number, timings (112/999 time, dispatch time, 112/999-hospital time), history, patient characteristics (age, gender), injuries, clinical findings (including (suspicion of) intoxication based on history and exam, first recorded HR, SBP, reactivity of pupils, GCS (including individual components), 12-lead ECG findings, interventions provided by ground ambulance- and HEMS crews, drugs administered, patient disposition (type of hospital), mode of transport, and (when performed/available) CT scan results. Computer aided dispatch (CAD) data were reviewed for all cases by one of the investigators (RdC), and compared to dispatch grades as noted in HEMSbase.

Interventions were subdivided into HEMS-specific and non-specific (other) interventions. For the purpose of the study, HEMS-specific interventions were defined as interventions that could only be provided by the HEMS crews such as Prehospital Emergency Anaesthesia (PHEA), administration of prothrombin complex concentrate (PCC, beriplex®), administration of intravenous (IV) antibiotics and/or antiviral drugs, hypertonic saline or IV vasopressors. Non-HEMS interventions recorded were placement of a supraglottic airway, IV/IO access, and administration of analgesics, anti-emetics, atropine, dextrose, or naloxone.

### Outcome measures

#### Primary outcome measure

HEMS-specific interventions performed in patients with unexplained LOC.

#### Secondary outcome measure

Clinical- and dispatch criteria related to HEMS interventions being performed.

### Ethics

This project met the National Research Ethics Service (NRES) definition of service evaluation audit (NRES, 2009) and therefore did not require ethical approval.

### Patient and public involvement

Patients and public were not involved in the design and conduct of the present study.

### Statistical analysis

Descriptive statistics are given as mean (SD) or median [IQR]. Comparisons across groups were made using Fisher’s exact test, Mann-Whitney U test and Student’s t-test, where appropriate. When three or more groups were present, nominal data were compared using Kruskall-Wallis test. Univariate correlation analysis with calculation of Spearman correlation coefficients was performed to evaluate the association of clinical- and treatment factors with the need for HEMS interventions on scene. Multivariable regression analysis including factors with a significant correlation (and an *r* > 0.2) was performed to determine which factors were independently related to HEMS interventions on scene. ROC analysis with calculation of AUC was performed to compare the diagnostic accuracy of different (combinations) of clinical variables to predict HEMS interventions in patients with a LOC. Missing values are reported in the results section of the manuscript according to the STROBE guideline [13]^.^ A *p*-value < 0.05 was regarded as statistically significant. All statistical analyses were conducted using SPSS 26.0 for Mac statistical package.

## Results

### Study population

**Fig. 1:**
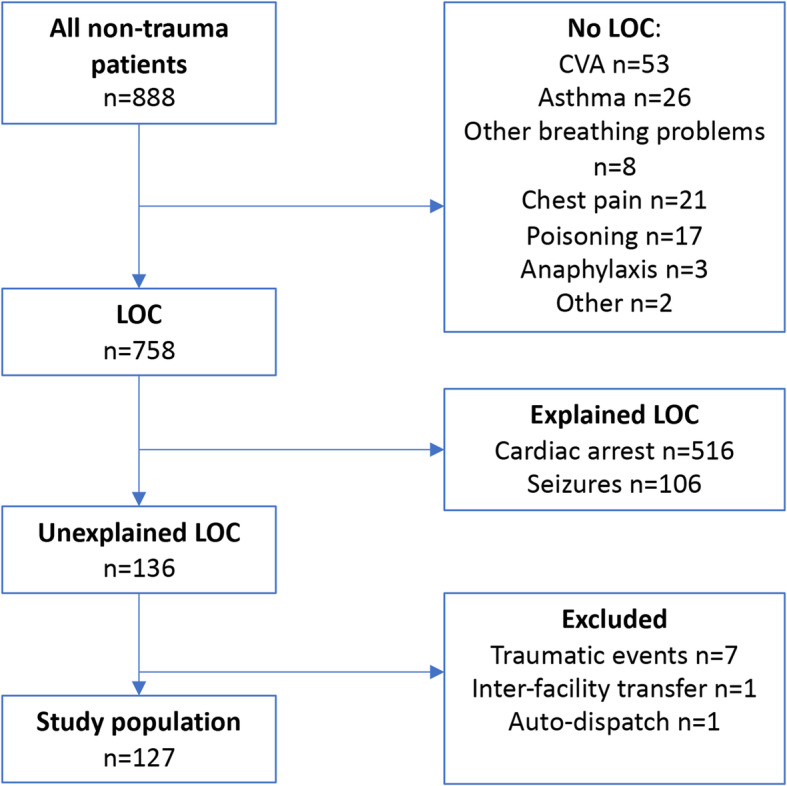
Study population

During the study period HEMS was dispatched to 888 non-trauma patients. Of these, 130 had no LOC. Of the 758 patients with LOC, cardiac arrest (*n* = 516) or seizures (*n* = 106) were identified as the cause of LOC at the moment of dispatch, and 136 patients with unexplained LOC. After chart review, 7 patients did have a traumatic event resulting in their collapse, 1 patient was an interfacility transfer, and 1 patient was attended after an auto-dispatch by the crew (Fig. 1). Further results refer to the remaining 127 patients.

### HEMS dispatch

HEMS was dispatched directly to 29 patients, either without (*n* = 6) or after (*n* = 23) interrogation by a HEMS dispatcher. Ninety-eight (74.8%) the of dispatches were on request of the ground ambulance crews (either direct requests or via the CCD). Based on the information provided by the 999-call takers in the CAD system, only in one of these 98 incidents the clinical picture could have triggered a direct dispatch. The remaining incidents were mainly described as “stroke”, “hypoglycaemia” or “general collapse”. As expected, the mean time from 112/999 call to arrival of the HEMS crew on scene was longer for crew requests compared to direct dispatch (14 [8–19] vs 39 [35–44] min, *p* < .001) (Table 1).

**Table 1:** Dispatch criteria

		N (%)	999-scene time (min)	*p*
Dispatch type				
*Direct*	Cat 1	6 (4.6)	5 (3)	<.001
	Cat 2	23 (17.6)	16 (14)	
*Request*	Cat 3	33 (25.2)	39 (18)	
	Cat 4	65 (49.6)	40 (23)	
Dispatch day	Weekday	95 (74.8)	32 (21)	.38
	Weekend	32 [25.2)	38 (28)	

Table 1**.** Displayed are mean (SD) 999-scene times. Cat 1; direct dispatch based on one grade 1 criterium; Cat 2, direct dispatch based on two grade 2 criteria; Cat 3, dispatch on request of critical care desk; Cat 4, dispatch on request of ambulance crew on scene.

Table 2 shows the patient characteristics of patients with unexplained LOC attended by HEMS stratified by type of dispatch. Mean age was 54 (17) years and the majority of the patients (60.8%) were male. 37 patients (31%) had profound hypertension on presentation, whereas hypotension was less frequently present (*n* = 3, 2.3%). GCS was still reduced in 106 (82%) of the patients upon arrival of HEMS, and 35 had one or two unresponsive pupils. Dysrhythmia’s and/or conduction abnormalities were found in 11 patients. In only a minority (11.0%) of the patients, intoxication was thought to play a role in the collapse as noted by the HEMS team.

**Table 2:** Patient characteristics of patients with a unexplained LOC attended by HEMS stratified by type of dispatch

	Whole group (*n* = 127)	Direct dispatch (*n* = 29)	Request dispatch (*n* = 98)	*p*
Age (years)	54 (17)	56 (18)	53 (16)	.49
Male (%)	60.8	69.0	58.4	.39
Witnessed	69 [52.7%]	19 [65.5%]	50 [51.0%]	.14
First HR (bpm)	86 (24)	82 (22)	87 (26)	.48
First SBP (mmHg)	149 (36)	149 (33)	148 (36)	.95
< 90	3 [2.3%]	1 [3.4%]	2 [2.0%]	.93
90–160	85 [66.1%]	20 [68.9%]	65 [66.3%]	
> 160	37 [29.9%]	7 [24.1%]	30 [30.6%]	
missing	2 [1.6%]	1 [3.4%]	1 [1.0%]	
First GCS	7 [4–14]	10 [4–14]	7 [4–13]	.15
14–15	34 [26%]	13 [44.8%]	21 [21.4%]	.095
8–13	26 [19.8%]	3 [10.3%]	23 [23.5%]	
3–8	65 [51.1%]	13 [44.5%]	52 [53.1%]	
missing	2 [1.5%]		2 [1.5%]	
Pupils				
Reactive (n)	92	23	69	.46
Unreactive (n)	35	6	29	
ECG				
Dysrhythmia (n)	11	0	11	.067
Other abnormalities (n)^a^	7	1	6	.69
Intoxication w alcohol and/or drugs (n)	14	1	13	.19
Hypoglycaemia (n)	3	0	3	.07

Table 2**.** Displayed are mean (SD) for continuous and median [IQR] for ordinal; variables. *HR* Heart rate, *SBP* Systolic blood pressure, *GCS* Glasgow Coma Scale. ^a^Other ECG abnormalities: anterior T wave inversion 1; STEMI 1; ST depression 1; LBBB 1, LV strain 1; VES 2.

### HEMS treatments

Table 3 shows the interventions performed on scene by ground ambulance and HEMS personnel. 119 patients (92%) had one or more non-HEMS interventions being performed on scene, whereas HEMS interventions were performed in 84 (65%) patients. PHEA was most often performed (*n* = 73, 56%) followed by the administration of hypertonic saline (*n* = 27, 21%), antibiotics/antiviral medication (*n* = 11, 8%) and vasopressor therapy (*n* = 8, 6%).

**Table 3:** Interventions provided by ground ambulance crew (non-HEMS interventions) and HEMS on scene in patients with unexplained LOC attended by HEMS

	Whole group (*n* = 127)	Direct dispatch (*n* = 29)	Request dispatch (*n* = 98)	*p*
**Non-HEMS interventions**				
Antiemetics	24	3	21	.28
Analgesia^a^	21	3	17	.41
Atropine	1	0	1	.99
Dextrose 10%	3	0	3	.59
Naloxone	7	1	6	.69
Supraglottic airway device	4	0	4	.57
IV	145	22	123	.016
IO	4 (HH) 5 (Tib)	3 (HH) 1 (Tib)	1(HH) 4(Tib)	.21
**HEMS interventions**				
PHEA	73	13	60	.088
Indication for PHEA				
Reduced GCS (n)	55	12	43	.29
Airway	10	0	10	
Compromise (n)				
Unmanageable (n)	7	1	6	
Resp failure (n)	1	0	1	
RSI regime:				
3–2-1^b^	59	8	46	1.00
1–1-1^c^	10	0	10	
Other	6	2	4	
Anticoagulant reversal	1	0	1	.99
Vasopressor therapy	8	1	7	.68
Antibiotics/ acyclovir	11	0	11	.067
Hypertonic saline	27	0	27	.007

Table 3**.** Displayed are numbers [%]. *IV* Intravenous, *IOI* Intraosseous, *PHEA* Prehospital Emergency Anesthesia, *LOC* Loss of consciousness, *HH* Humeral head, *Tib* Tibia,. ^a^ Analgesia: fentanyl (*n* = 2), Morphine (*n* = 2), Ketamine (*n* = 3), and paracetamol (*n* = 14). ^b^ Fentanyl 3 mcg/kg, Ketamine 2 mg/kg and Rocuronium 1 mg/kg. c Fentanyl 1 mcg/kg, Ketamine 1 mg/kg and Rocuronium 1 mg/kg HR.

### Dispatch- and patient characteristics in relation to HEMS interventions and patient disposition

When HEMS was sent directly (Grade 1, *n* = 6), no patients received an RSI and only one patient was conveyed to hospital. When HEMS was sent after interrogation by a HEMS dispatcher (Grade 2, *n* = 23), 16 patients were conveyed, and 13 received an RSI. When a crew request (either direct or via the CCD) triggered a HEMS response, (Grades 3 or 4, *n* = 98), 84 patients were conveyed, and 60 patients received an PHEA. Hyperosmolar therapy (Hypertonic saline 5%) and antibiotic/antiviral drugs were only administered in the dispatch- after-request group.

Univariate correlation analysis revealed that GCS (*r* = − 0.6, *p* < .001) and SBP (*r* = 0.31, *p* < .001) both showed an association with HEMS interventions being performed on scene. In multivariate analysis, GCS and initial SBP were independent predictors of the need for HEMS interventions. ROC analysis with various cut-off values for SBP and GCS revealed that the highest AUC was obtained for GCS < 13 (AUC 0.85, sensitivity 94.9%, specificity 75%). Addition of SBP (GCS < 13 and/or SBP > 160mmHG) did not improve the AUC (0.84, sensitivity 97.5%, specificity 70.5%). Of the patients who had an initial GCS ≥13 (*n* = 40), 2 required an RSI in their clinical course, and 2 received antibiotics for suspected meningitis, whereas 27 patients with a GCS < 13 did not require any HEMS interventions.

Most patients seen by HEMS with unexplained LOC were triaged to a major trauma centre with neurosurgical facilities and were escorted by HEMS either by road (ground escort - GE) or by helicopter (carry), Table 4. The average time from 112/999 call to arrival in hospital was 130 (34) minutes. Patients seen after a crew request showed a trend towards slightly longer 999 to hospital time. In 49 patients (39% of all patients) a CT-scan made shortly after admission revealed gross pathology explaining the LOC. Most frequent findings were subarachnoid (*n* = 25), and intra-parenchymal- (*n* = 21) haemorrhages. Traumatic findings (likely as a result of the collapse) were reported for two patients (1 epidural haematoma, 1 cerebral contusion and skull fracture).

**Table 4:** Patient disposition and outcome of patients attended by HEMS with unexplained LOC

	Whole group (*n* = 127)	Direct dispatch (*n* = 29)	Crew request (n = 98)	p
Transport				
GA	29	13	16	.002
GE	56	11	45	
Aircraft carry	42	5	37	
Disposition				
TU	43	14	29	.044
MTC	84	15	69	
Time to hospital	130 (34)	118 (32)	133 (34)	.095
CT-head (n)				
Positive	49	10	39	.90
Negative	28	7	21	
Not performed or unavailable	53	12	38	

Table 4**.** Displayed are numbers (n) and mean (SD) 999-hospital times. *GA* Ground assist, *GE* Ground escort, *TU* Trauma unit, *MTC* Major trauma centre.

## Discussion

In this study, we report that HEMS dispatchers in collaboration with ambulance personnel are able to select a subset of patients with unexplained LOC with a high prevalence of acute neurological pathology, who might benefit from HEMS specific interventions such as prehospital RSI, vasopressor therapy and early antibiotic administration. Presenting GCS can be used to inform this selection process at an early stage. This are new findings, as we are not aware of any previous studies that have been published about HEMS dispatch to non-trauma (neuro) cases.

Many patients in this study suffered from acute neurological pathology, in particular spontaneous intracranial bleeds. These patients are best served by early prevention of hypoxia, hypercarbia and hypotension, in order to preserve cerebral perfusion and prevent secondary brain injury [14]. Prevention of hypertension, with the risk of intracranial haematoma expansion and/or rebleeding, is of equal importance. HEMS teams have knowledge and skills beyond standard ground ambulance crews to treat these patients. Furthermore, HEMS may provide a mode of expedited transport to a neurosurgical facility. Previous studies on patients suffering from aneurysmal subarachnoid haemorrhage highlight the potential ‘time-saving’ nature of such a helicopter transport to a tertiary centre [15]. Finally, the presence of a critical care team may add value by senior decision making, not only regarding treatment, but also regarding mode of transport and ultimate disposition.

In our study, HEMS-specific interventions were performed in 65% of all patients attended. RSI was the most frequently performed intervention (56%), but other treatments to prevent secondary brain injury (such as hyperosmolar and/or vasopressor therapy) were also commonly provided. Whether or not the interventions provided had a positive effect on oxygenation, cerebral perfusion or (neurological intact) survival of our patients was beyond the scope of this study, as we did not have access to a matched control group of patients who were not attended by HEMS. However, previous studies performed in patients with traumatic brain injury demonstrated that HEMS involvement reduced the incidence of hypoxia and airway complications in patients with traumatic brain injury [16].

HEMS teams were dispatched directly in only a minority of the patients with LOC in our cohort. Although robust evidence-based HEMS tasking criteria are available for trauma patients [6, 10], service deployment to non-trauma patients is often less guided by strict criteria [17, 18]. Historically, the dispatch process in many EMS systems for non-trauma relies on a ‘chief complaint’ creating a cascade of actions and subsequent dispatch of EMS resource. However, as HEMS is a scarce resource, it can only respond to a small percentage of all patients presenting with a collapse with LOC. Over-triage of these patients increases sensitivity (no patients requiring HEMS interventions are missed), but significantly impacts specificity (many unnecessary dispatches), whilst under triage results in the opposite. Undertriage not only carries the risk of suboptimal treatment, but also of more patients being referred to centres without neurosurgical facilities, with a subsequent need for interfacility transport and a delay in definitive care [11]. With only 127 patients attended with unexplained LOC in 3.5 years (1.8% of > 7000 patients in total attended), it is likely that more patients could have benefitted from HEMS during the study period. Dispatch criteria should therefore be continuously calibrated [17, 19]. Our findings may help to improve dispatch accuracy, as we demonstrate that patients with a GCS ≥ 13 seldom need HEMS interventions. However, no combination of clinical criteria alone is perfect. Novel approaches focussing on communication in the initial call [20] and live video transmission from scene [21] may help to identify patients needing HEMS interventions earlier.

For the majority of the patients (75%), HEMS was dispatched on request of the ground ambulance crews on scene. This inevitably led to a delayed dispatch and a trend towards a longer 112/999-to-hospital time. Early clinical decision-making regarding triage and the need for HEMS assistance in paramedic practice for patients with LOC is therefore important [22]. This is stressed by the finding that HEMS interventions were as commonly performed in patients attended after crew request as in patients where HEMS was dispatched directly. When HEMS assistance is deemed required, ground ambulance crews are encouraged to perform critical interventions, such as gaining IV access before the arrival of HEMS to expedite subsequent HEMS interventions as PHEA, and thereby to minimize the time to definitive care.

Our study has several limitations, some of them being inherent to the retrospective design. First, we had to rely on the data as provided by the HEMS teams. Although there were some missing data, overall data completeness was good due to the use of our electronic patient record with dedicated data entry fields for all patients. Second, HEMS only attends a fraction of all patients with LOC. Although we demonstrate that dispatchers and ambulance crews were able to select a cohort of patients who most likely benefited from HEMS interventions, we don’t know how many patients were *not* attended during the study period who might have benefited from HEMS as well. Furthermore, care should be taken to extrapolate our findings to other services. Dispatch criteria for non-trauma dispatch may vary between services, and this may affect the subgroup of patients with LOC attended by HEMS. Finally, early advanced HEMS interventions are not necessarily related to an improved outcome even though this is likely from a physiological point of view, as HEMS involvement may have negative side effects as well (such as extended scene times), and in some instances, no intervention will be the best intervention.

## Conclusion

HEMS dispatchers and ambulance personnel are able to identify a cohort of patients with unexplained LOC of medical origin who suffer from potentially life threatening (mainly neurological) pathology, in whom HEMS specific intervention are frequently required. Presenting GCS can be used to inform the triage process of patients with LOC at an early stage.

## Supplementary Information

**Additional file 1:** **Supplementary file 1** HEMS tasking criteria AAKSS version 2.

## Data Availability

The datasets used and/or analysed during the current study are available from the corresponding author on reasonable request.
