# Supplementary figures and images for: Dispatch of a helicopter emergency medicine service to patients with a sudden, unexplained loss of consciousness of medical origin
Source: BMC Emerg Med. 2020 Nov 25;20:92. doi: 10.1186/s12873-020-00388-x (PMC7690130; doi:10.1186/s12873-020-00388-x)

**Supplementary file 1** HEMS tasking criteria AAKSS version 2


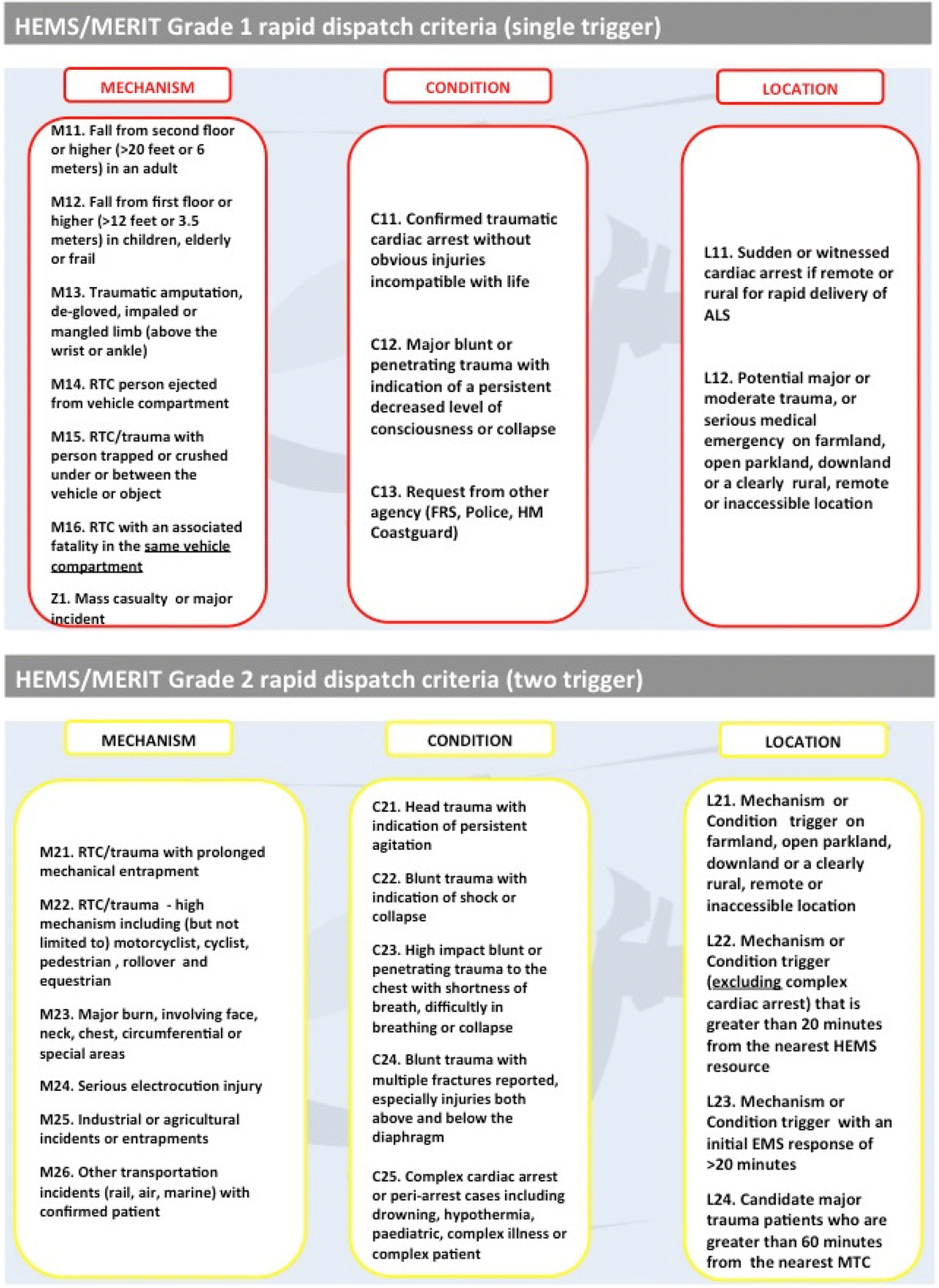

Supplement: Supplementary file 1 — Additional file 1: Supplementary file 1 HEMS tasking criteria AAKSS version 2. [file 12873_2020_388_MOESM1_ESM.docx]
